# Supplementary material for: Exploring multistability in prismatic metamaterials through local actuation
Source: Nat Commun. 2019 Dec 6;10:5577. doi: 10.1038/s41467-019-13319-7 (PMC6898700; doi:10.1038/s41467-019-13319-7)
Supplement: Supplementary file 1 — Supplementary Information [file 41467_2019_13319_MOESM1_ESM.pdf]

Supplementary Information for

Exploring multistability in prismatic metamaterials through local actuation

Iniguez-Rabago et al.

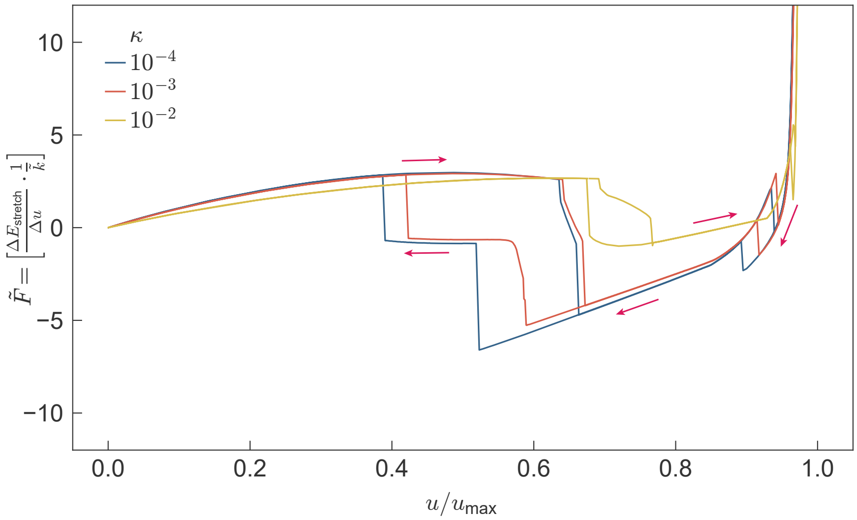

Supplementary Figure 1. Normalized force-displacement response obtained from simulated compression tests. The response was obtained for a prismatic structure based on a cuboctahedron using different ratios between hinge stiffness and face stiffness. The behavior of the simulations is qualitatively similar for $\kappa = 10^{-3}$ and $\kappa = 10^{-4}$, both showing two instabilities when compressing the structure and three when returning to the initial configuration. Although not shown here, similar results are obtained for $\kappa < 10^{-4}$. However, when $\kappa = 10^{-2}$, the response changes, and only two instabilities occur during unloading. Furthermore, the minimum force increases, reducing the stability of the stable state observed for $\tilde{F} < 0$.

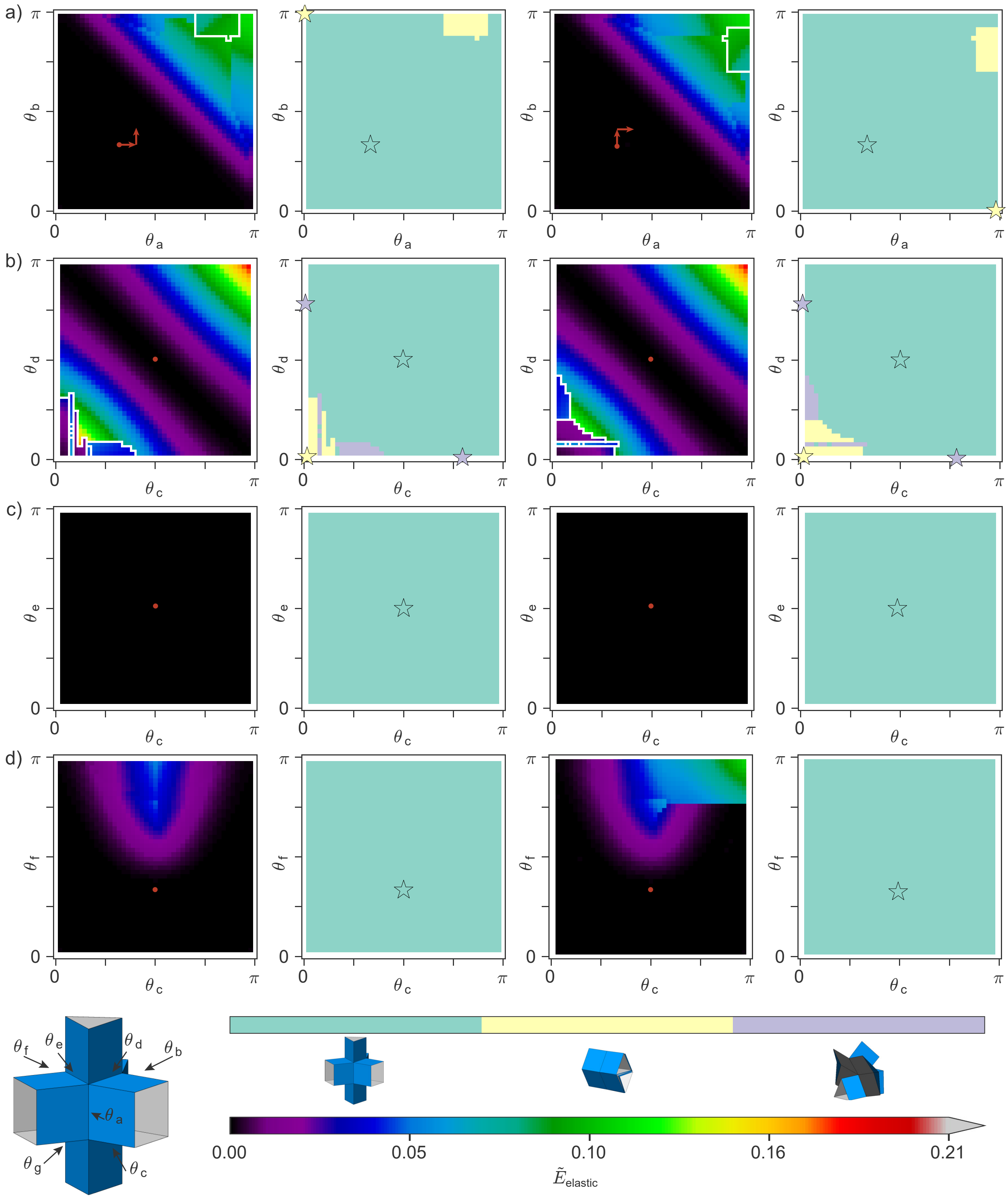

Supplementary Figure 2. Projected energy landscapes and state diagrams for a prismatic structure based on a triangular prism. Here, we fold the structure in two steps by applying a torque to two hinges in sequence. Different projections of the energy landscape are obtained by loading the structure along a) θ_{b} and θ_{a}, b) θ_{d} and θ_{c}, c) θ_{e} and θ_{c}, d) θ_{f} and θ_{c}, e) θ_{a} and θ_{c}, and f) θ_{g} and θ_{c}. We record the elastic energy of the final state, and repeat this process for all possible combinations of the angle values. The results are then presented as a 2D projection of the energy landscape. To show the dependency of the loading path on the final configuration, we also show the results obtained when changing the order of loading. Moreover, we relax the prismatic structure from the folded configurations to reach the local minima and obtain a state diagram. The star symbols indicate the values of the angles of these minima, where points in the plot relax to a star symbol with the same color.

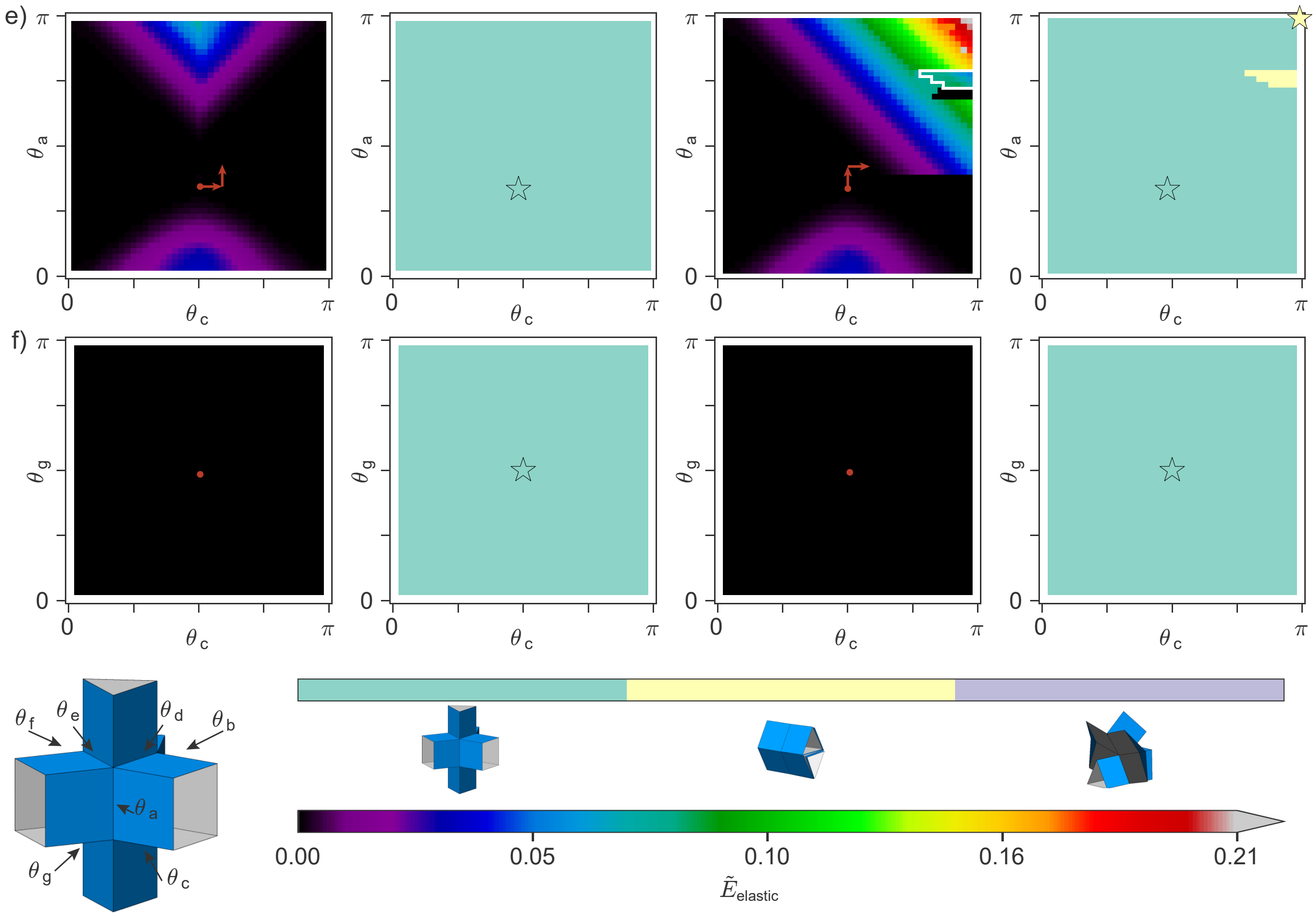

Supplementary Figure 2. (continued) **Projected energy landscapes and state diagrams for a prismatic structure based on a triangular prism.** Here, we fold the structure in two steps by applying a torque to two hinges in sequence. Different projections of the energy landscape are obtained by loading the structure along a) $\theta_{\rm b}$ and $\theta_{\rm a}$, b) $\theta_{\rm d}$ and $\theta_{\rm c}$, c) $\theta_{\rm e}$ and $\theta_{\rm c}$, d) $\theta_{\rm f}$ and $\theta_{\rm c}$, e) $\theta_{\rm a}$ and $\theta_{\rm c}$, and f) $\theta_{\rm g}$ and $\theta_{\rm c}$. We record the elastic energy of the final state, and repeat this process for all possible combinations of the angle values. The results are then presented as a 2D projection of the energy landscape. To show the dependency of the loading path on the final configuration, we also show the results obtained when changing the order of loading. Moreover, we relax the prismatic structure from the folded configurations to reach the local minima and obtain a state diagram. The star symbols indicate the values of the angles of these minima, where points in the plot relax to a star symbol with the same color.

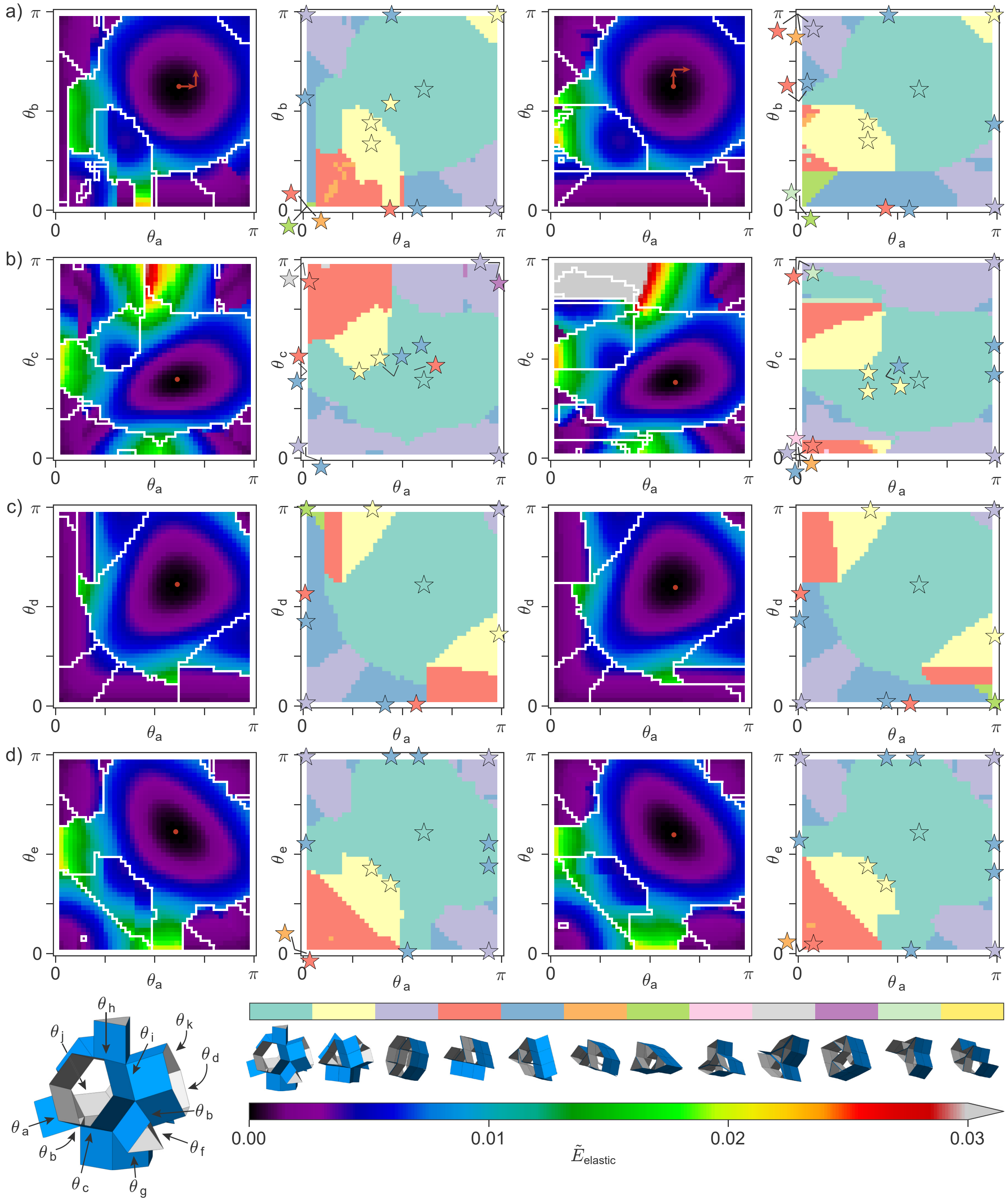

Supplementary Figure 3. Projected energy landscapes and state diagrams for a prismatic structure based on a truncated tetrahedron. Here, we fold the structure in two steps by applying a torque to two hinges in sequence. Different projections of the energy landscape are obtained by loading the structure along a) θ_b and θ_a, b) θ_c and θ_a, c) θ_d and θ_a, d) θ_e and θ_a, e) θ_f and θ_a, f) θ_g and θ_a, g) θ_h and θ_a, h) θ_i and θ_a, i) θ_j and θ_a, j) θ_f and θ_c, and k) θ_k and θ_c. We record the elastic energy of the final state, and repeat this process for all possible combinations of the angle values. The results are then presented as a 2D projection of the energy landscape. To show the dependency of the loading path on the final configuration, we also show the results obtained when changing the order of loading. Moreover, we relax the prismatic structure from the folded configurations to reach the local minima and obtain a state diagram. The star symbols indicate the values of the angles of these minima, where points in the plot relax to a star symbol with the same color.

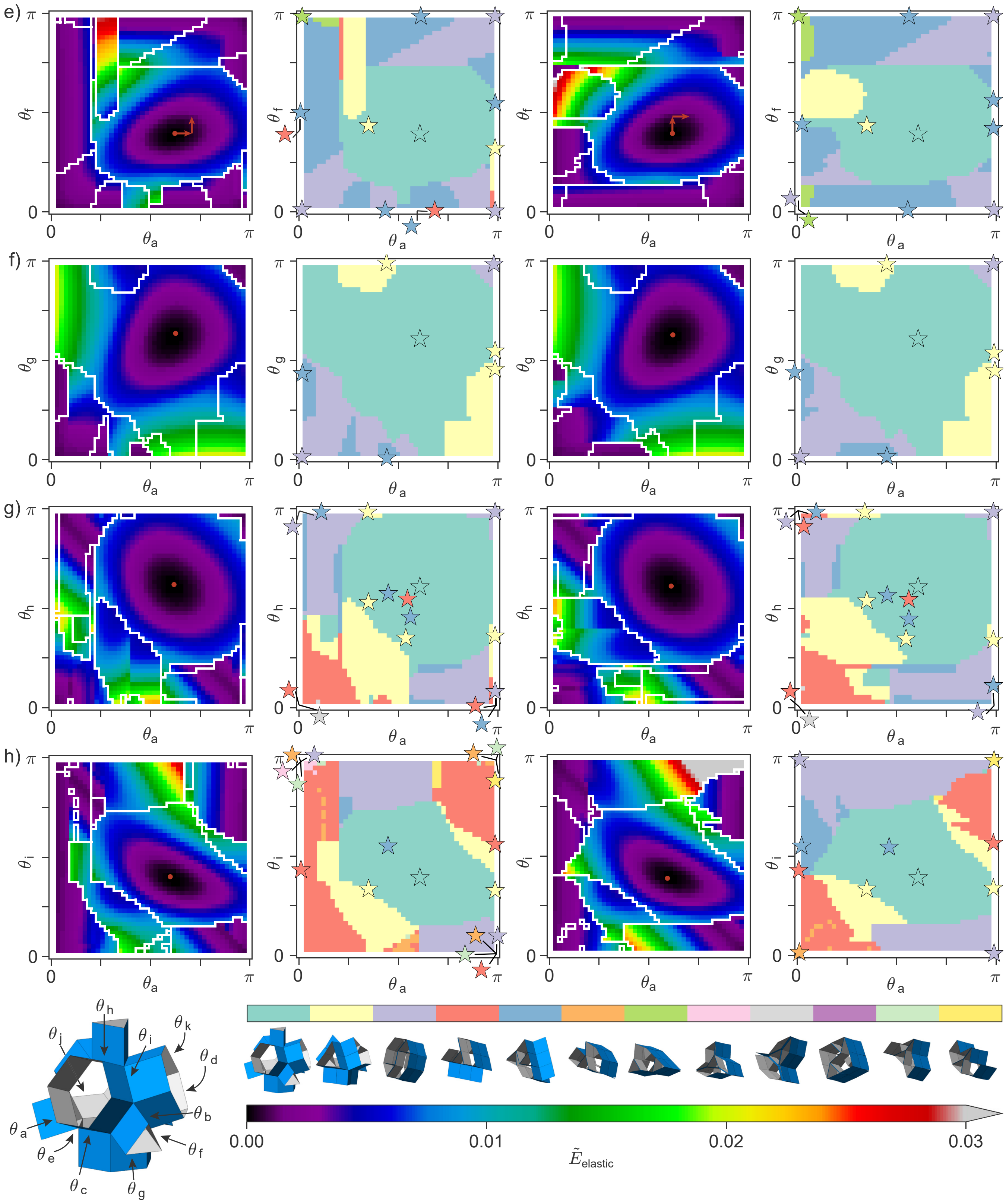

Supplementary Figure 3. (continued) **Projected energy landscapes and state diagrams for a prismatic structure based on a truncated tetrahedron.** Here, we fold the structure in two steps by applying a torque to two hinges in sequence. Different projections of the energy landscape are obtained by loading the structure along a) θ_b and θ_a, b) θ_c and θ_a, c) θ_d and θ_a, d) θ_e and θ_a, e) θ_f and θ_a, f) θ_g and θ_a, g) θ_h and θ_a, h) θ_i and θ_a, i) θ_j and θ_a, j) θ_f and θ_c, and k) θ_k and θ_c. We record the elastic energy of the final state, and repeat this process for all possible combinations of the angle values. The results are then presented as a 2D projection of the energy landscape. To show the dependency of the loading path on the final configuration, we also show the results obtained when changing the order of loading. Moreover, we relax the prismatic structure from the folded configurations to reach the local minima and obtain a state diagram. The star symbols indicate the values of the angles of these minima, where points in the plot relax to a star symbol with the same color.

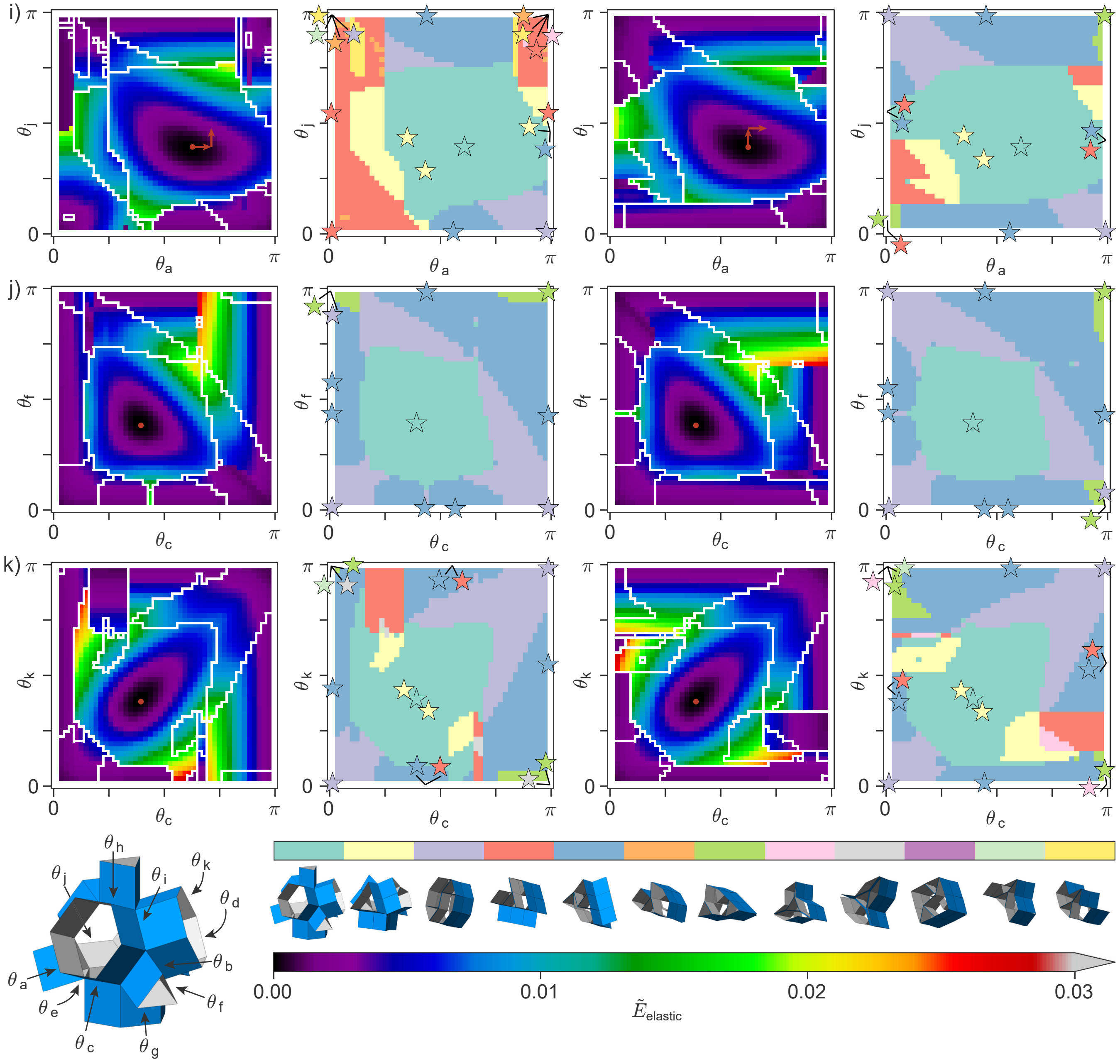

Supplementary Figure 3. (continued) **Projected energy landscapes and state diagrams for a prismatic structure based on a truncated tetrahedron.** Here, we fold the structure in two steps by applying a torque to two hinges in sequence. Different projections of the energy landscape are obtained by loading the structure along a) θ_b, and θ_a, b) θ_c and θ_a, c) θ_d and θ_a, d) θ_e and θ_a, e) θ_f and θ_a, f) θ_g and θ_a, g) θ_h and θ_a, h) θ_i and θ_a, i) θ_j and θ_a, j) θ_f and θ_c, and k) θ_k and θ_c. We record the elastic energy of the final state, and repeat this process for all possible combinations of the angle values. The results are then presented as a 2D projection of the energy landscape. To show the dependency of the loading path on the final configuration, we also show the results obtained when changing the order of loading.Moreover, we relax the prismatic structure from the folded configurations to reach the local minima and obtain a state diagram. The star symbols indicate the values of the angles of these minima, where points in the plot relax to a star symbol with the same color.

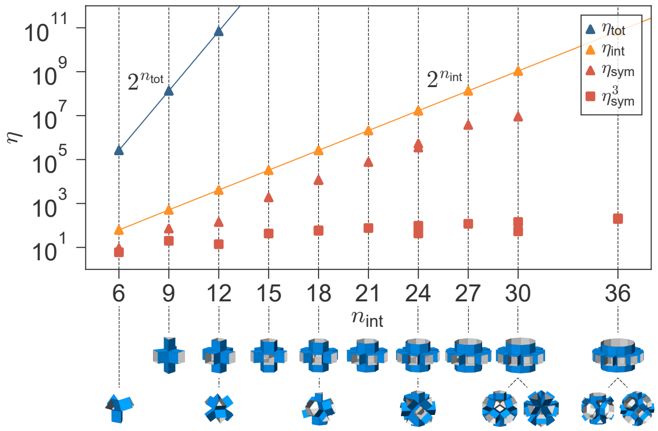

Supplementary Figure 4. Total number of possible hinge combinations for various prismatic structures. Starting from considering both internal and external hinges (blue triangles), the amount of combinations can be reduced by only considering the internal hinges (yellow triangles). Further reductions can be made by using our custom algorithm based on directed graphs to remove symmetric combinations (red triangles), and by only considering combinations up to three hinges (red squares).

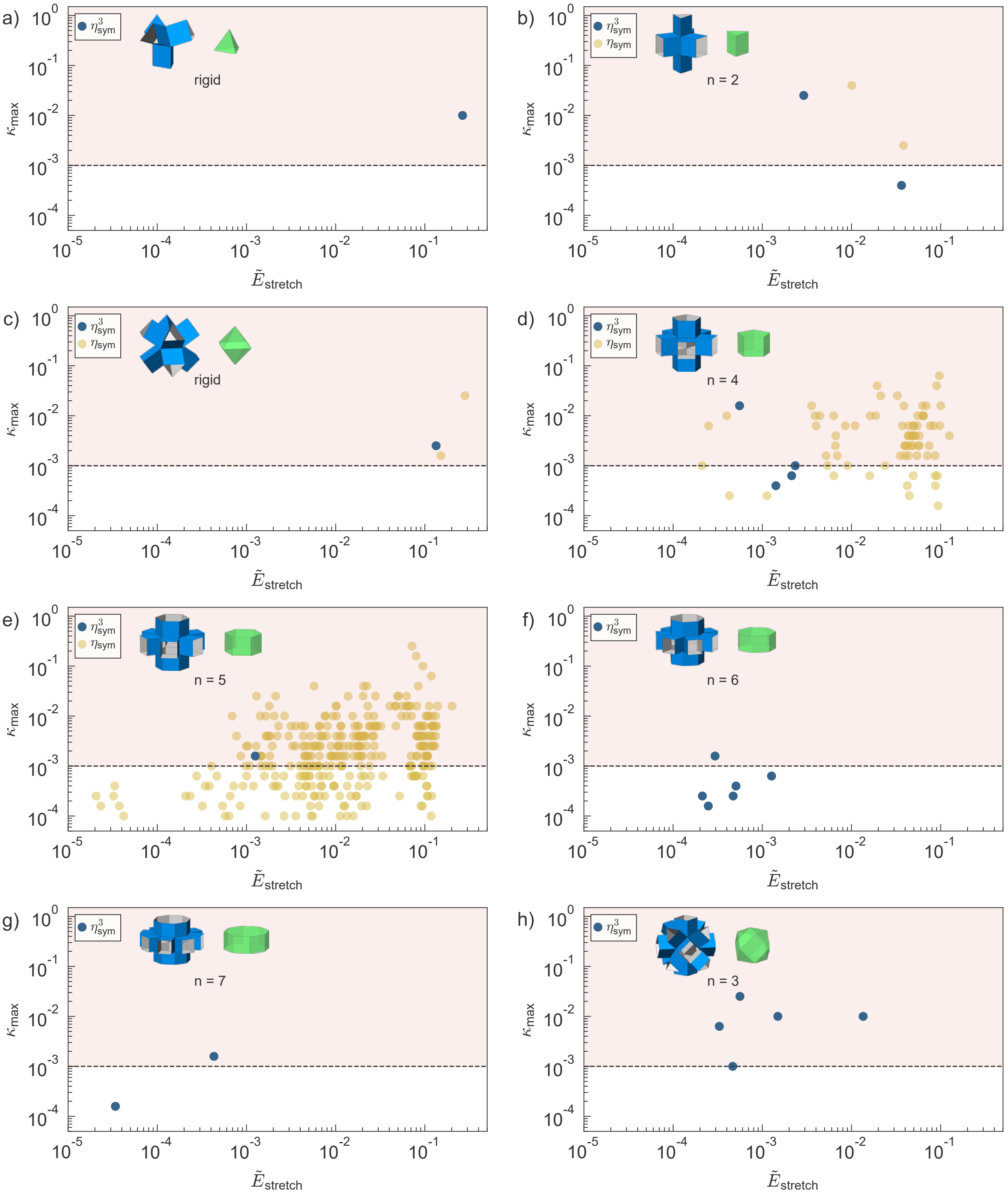

Supplementary Figure 5. Normalized stretch energy ($\tilde{E}_{\mathrm{stretch}}$) and maximum stiffness ratio ($\kappa_{\max}$) for multiple prismatic structures. Here we only show the unique stable states found with our numerical algorithm. Additionally we mention the amount of degrees of freedom (n) for each structure or if it is rigid when assuming rigid faces, adapted from [1]. For prismatic structures based on a a) tetrahedron, b) triangular prism, c) octahedron, d) pentagonal prism, and e) hexagonal prism, we considered all unique hinge combinations to find stable states, while for prismatic structures based on a f) heptagonal prism g) octagonal prism, h) cuboctahedron, i) nonagonal prism, j) decagonal prism, k) dodecahedron, l) dodecagonal prism, m) truncated cube, n) truncated octahedron, o) rhombicuboctahedron, and p) truncated cuboctahedron, we considered combinations up to a maximum of three hinges.

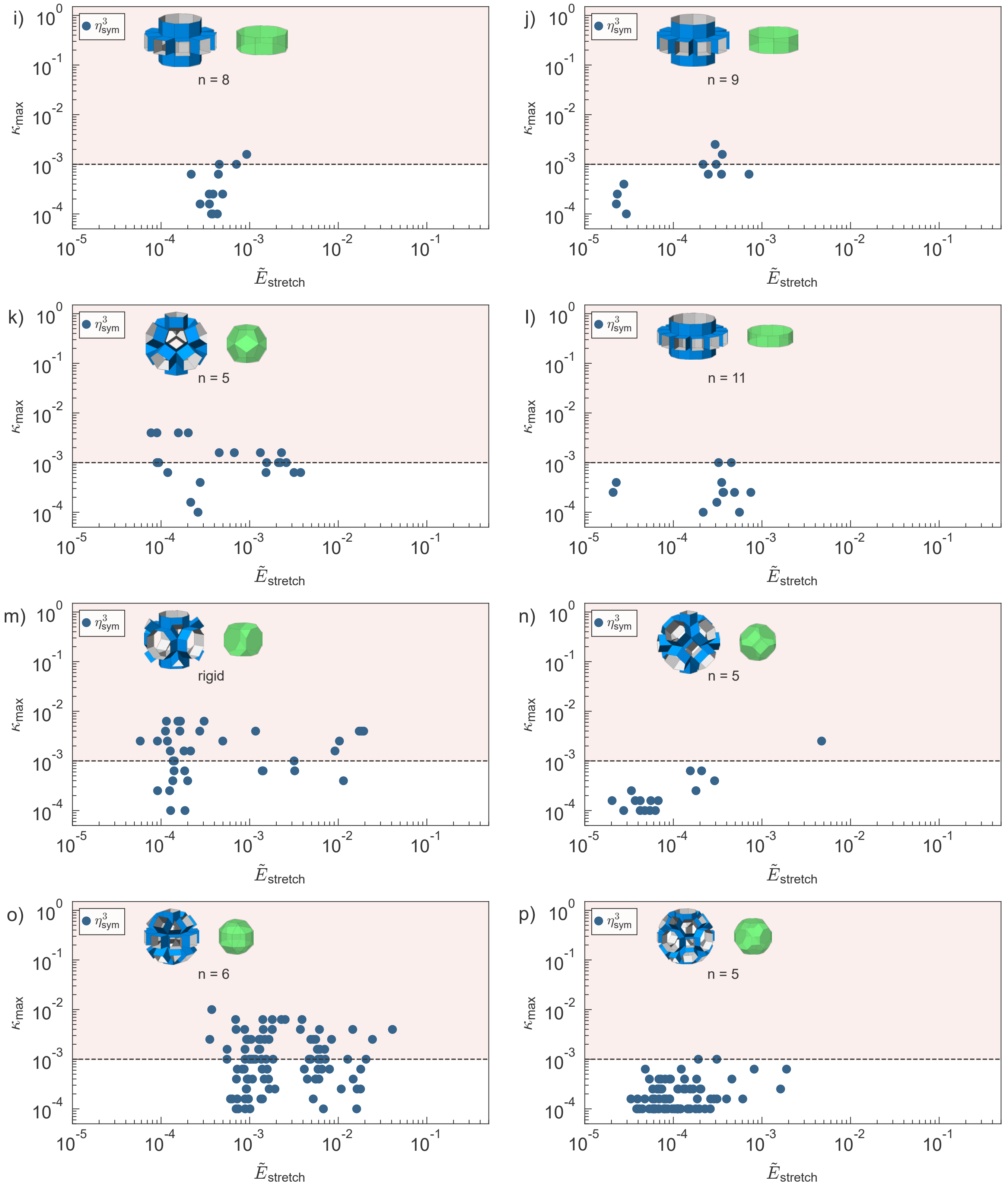

Supplementary Figure 5. (continued) **Normalized stretch energy ($\tilde{E}_{\mathrm{stretch}}$) and maximum stiffness ratio (κ_{max}) for multiple prismatic structures.** Here we only show the unique stable states found with our numerical algorithm. Additionally we mention the amount of degrees of freedom (n) for each structure or if it is rigid when assuming rigid faces, adapted from [1]. For prismatic structures based on a a) tetrahedron, b) triangular prism, c) octahedron, d) pentagonal prism, and e) hexagonal prism, we considered all unique hinge combinations to find stable states, while for prismatic structures based on a f) heptagonal prism g) octagonal prism, h) cuboctahedron, i) nonagonal prism, j) decagonal prism, k) dodecahedron, l) dodecagonal prism, m) truncated cube, n) truncated octahedron, o) rhombicuboctahedron, and p) truncated cuboctahedron, we considered combinations up to a maximum of three hinges.

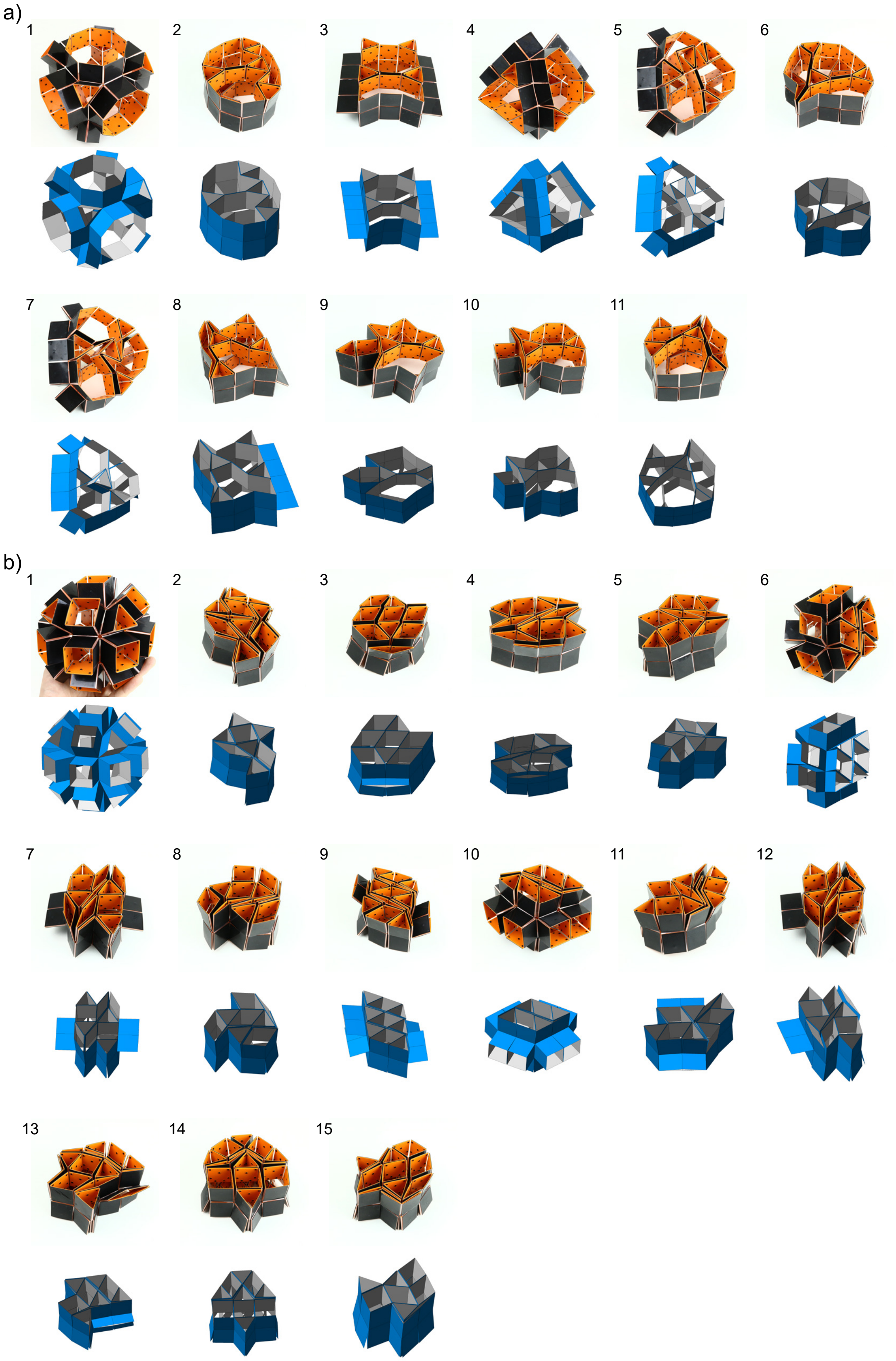

Supplementary Figure 6. Comparison of selected stable states found with our numerical algorithm and experiments. We show this comparison for two prismatic structures based on a) a truncated cube and b) a rhombicuboctahedron.

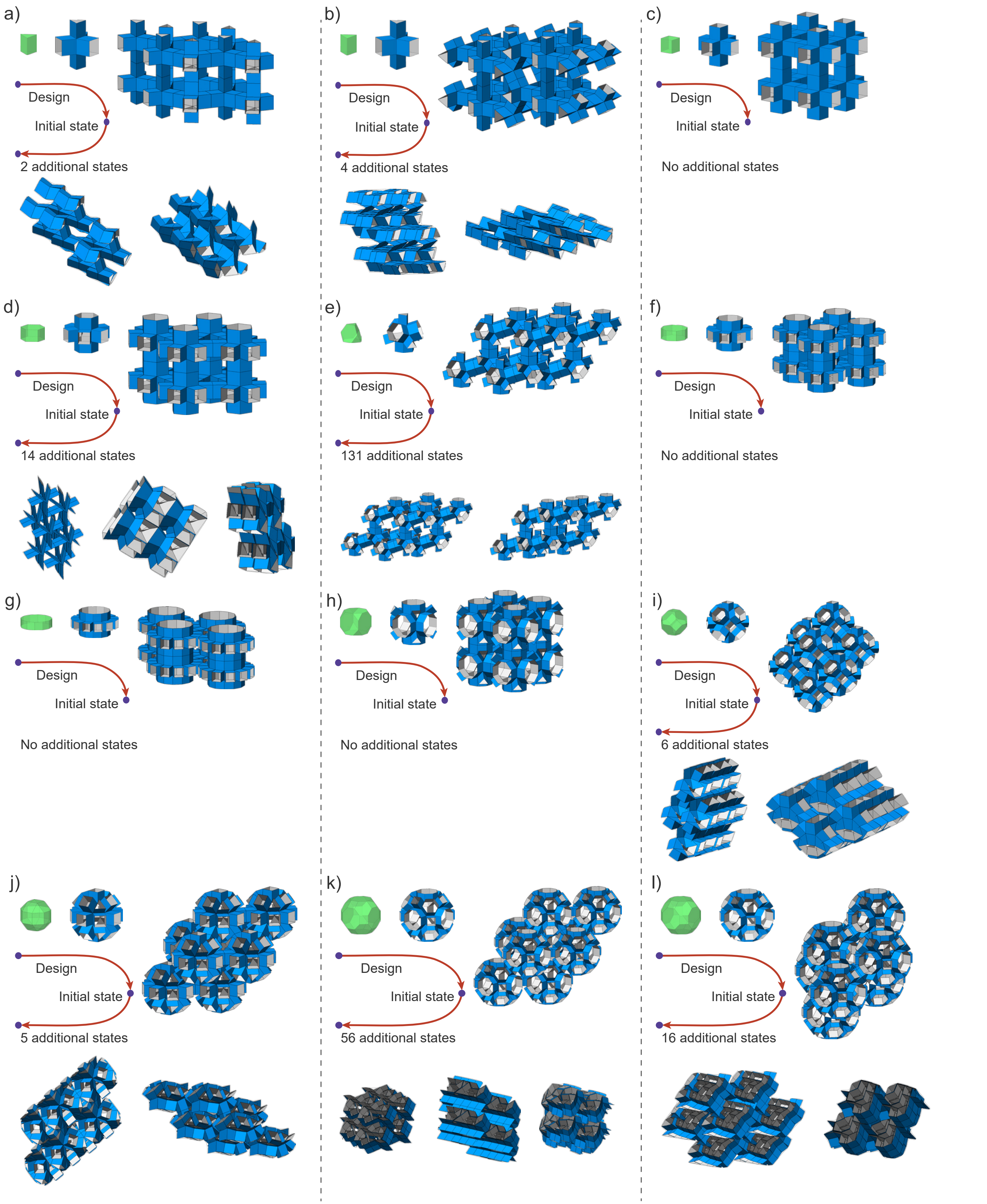

Supplementary Figure 7. Mechanical metamaterials based on cubic tessellations of prismatic structures and their unique stable states. These metamaterials are assembled from prismatic structures based on a) and b) triangular prisms, c) cubes, d) hexagonal prisms, e) truncated tetrahedrons, f) octagonal prisms, g) dodecagonal prisms, h) truncated cubes, i) truncated octahedrons, j) rhombicuboctahedrons, and k) and l) truncated cuboctahedrons. For most of the metamaterials a selection of the obtained stable states are shown.

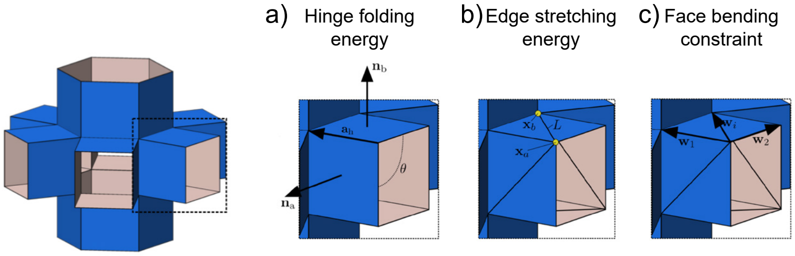

Supplementary Figure 8. Schematic of the extruded unit cell [1].

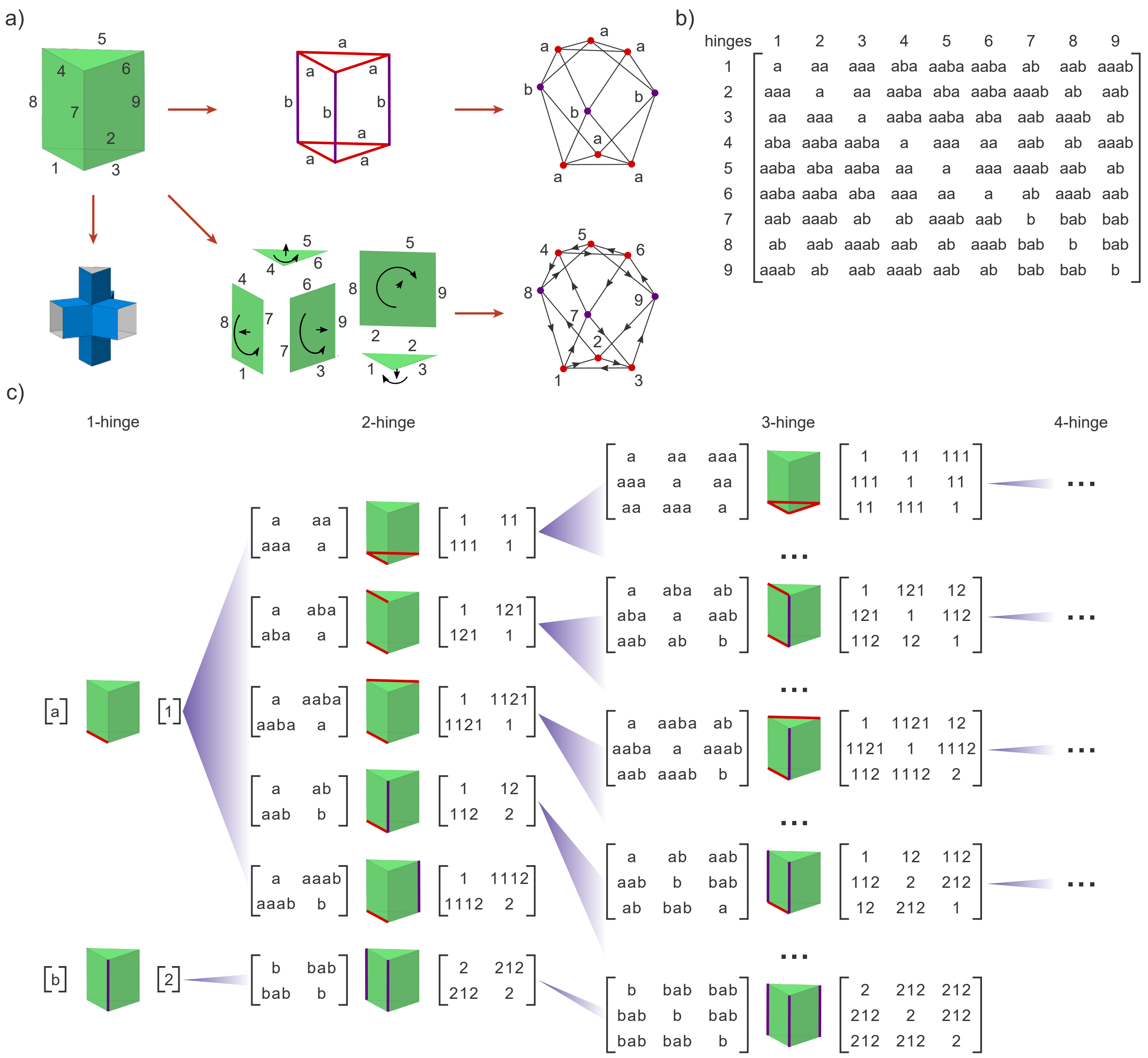

Supplementary Figure 9. Reduction of the hinge selection by exploiting the symmetries of a prismatic structure considering only the internal hinges. a) Starting from the triangular prism that forms the basis for a prismatic structure, we number the edges and give them types depending on the connected faces. We assign a node to each edge with the same type and connect these nodes depending on their common vertices in the polyhedron to construct a graph. Additionally, to assign directions to the connections in the graph, we apply the right-hand rule to its normal. b) We determine the minimum distance between nodes. Note that instead of using the distance, we denote the path by the type of the nodes that are encountered when traveling along the path. c) We extract all principal sub-matrices that represent the hinge selections. We replace the node type by a numerical number in order to obtain the eigenvalues of the sub-matrices. In this step, symmetric hinge selections are removed by removing those that have sub-matrices with the same eigenvalues as already selected hinge combinations.

Supplementary References

1. Overvelde JT, Weaver JC, Hoberman C, Bertoldi K (2017) Rational design of reconfigurable prismatic architected materials. *Nature* 541(7637):347–352.
