# Supplementary material for: Exploring multistability in prismatic metamaterials through local actuation
Source: Nat Commun. 2019 Dec 6;10:5577. doi: 10.1038/s41467-019-13319-7 (PMC6898700; doi:10.1038/s41467-019-13319-7)
Supplement: Supplementary file 2 — Description of Additional Supplementary Files [file 41467_2019_13319_MOESM2_ESM.docx]

Description of Additional Supplementary Files

**Supplementary Movie 1.** Experimental and numerical compression test. We perform a comparison between an experimental and numerical compression test applied to a prismatic structure based on a cuboctahedron. The simplified model used in simulations shows good qualitative agreement with experiments performed on a prototype made from 3D printed faces (PLA) connected by flexible hinges (Mylar).

**Supplementary Movie 2.** Projected energy landscape and state diagram. We select three folding sequences and project them on the energy landscape of a prismatic structure based on a triangular prism. To obtain each folding sequence we fold two hinges consecutively, after which we let the structure relax to a stable state. By repeating this approach, we find a state diagram of the stable states that can be achieved.

**Supplementary Movie 3.** Stable States of prismatic structures. We verify the stable states found for the prismatic structures based on a truncated tetrahedron and a cube. While many of the stable states of the prismatic structure based on a truncated tetrahedron can be achieved using a prototype with Mylar hinges, some states are not stable and relax to other configurations. Furthermore, for the prismatic structure based on a cube most of the stable states can only be achieved when replacing the Mylar hinges with more stretchable silicon hinges.

**Supplementary Movie 4.** Multistable metamaterials. We show the results of our numerical method applied to multistable metamaterials that are based on cubic tessellations of prismatic structures based on a cuboctahedron and a rhombicuboctahedron. The simulation of the metamaterial’s behavior was performed by applying periodic boundary conditions to the individual building block in addition to the local actuation patterns that previously resulted in stable configurations for the unit cell.

**Supplementary Movie 5.**Varying the multistable behavior in a metamaterial. We control the multistability of a metamaterial based on a cuboctahedron by adjusting κ. In experiments, this can be achieved by changing the hinge thickness. When we build our prototypes with Mylar hinges with a thickness of 50 µm, the metamaterial exhibits multiple stable states. However, when building the material with 125 µm thick Mylar hinges it is no longer multistable.

**Supplementary Movie 6.**Pneumatic actuation of a prismatic structure. We apply pneumatic actuation to a prismatic structure based on a truncated tetrahedron. We use pneumatic pouches to actuate two hinges of the structure individually, and are able to deform the structure into four stable states.
